# Untargeted lipidomic features associated with colorectal cancer in a prospective cohort

**DOI:** 10.1186/s12885-018-4894-4

**Published:** 2018-10-19

**Authors:** Kelsi Perttula, Courtney Schiffman, William M B Edmands, Lauren Petrick, Hasmik Grigoryan, Xiaoming Cai, Marc J Gunter, Alessio Naccarati, Silvia Polidoro, Sandrine Dudoit, Paolo Vineis, Stephen M Rappaport

**Affiliations:** 1 School of Public Health, University of California, Berkeley, CA 94720-7356 USA; 2 California Institute for Quantitative Biosciences, University of California, Berkeley, CA 94720 USA; 3 International Agency for Research on Cancer, Lyon, France; 4 Italian Institute for Genomic Medicine (IIGM), Torino, Italy; 5 MRC-PHE Centre for Environment and Health, Imperial College, Place London W2 1PG, Norfolk, UK; 6 Department of Statistics, University of California, Berkeley, CA 94720 USA; 7 Department of Environmental Medicine and Public Health, Icahn School of Medicine at Mount Sinai, New York, New York, 10029 USA

**Keywords:** Colorectal cancer, Lipidomics, Metabolomics, EPIC, Untargeted, Biomarkers

## Abstract

**Background:**

Epidemiologists are beginning to employ metabolomics and lipidomics with archived blood from incident cases and controls to discover causes of cancer. Although several such studies have focused on colorectal cancer (CRC), they all followed targeted or semi-targeted designs that limited their ability to find discriminating molecules and pathways related to the causes of CRC.

**Methods:**

Using an untargeted design, we measured lipophilic metabolites in prediagnostic serum from 66 CRC patients and 66 matched controls from the European Prospective Investigation into Cancer and Nutrition (Turin, Italy). Samples were analyzed by liquid chromatography-high-resolution mass spectrometry (LC-MS), resulting in 8690 features for statistical analysis.

**Results:**

Rather than the usual multiple-hypothesis-testing approach, we based variable selection on an ensemble of regression methods, which found nine features to be associated with case-control status. We then regressed each selected feature on time-to-diagnosis to determine whether the feature was likely to be either a potentially causal biomarker or a reactive product of disease progression (reverse causality).

**Conclusions:**

Of the nine selected LC-MS features, four appear to be involved in CRC etiology and merit further investigation in prospective studies of CRC. Four other features appear to be related to progression of the disease (reverse causality), and may represent biomarkers of value for early detection of CRC.

**Electronic supplementary material:**

The online version of this article (10.1186/s12885-018-4894-4) contains supplementary material, which is available to authorized users.

## Background

Colorectal cancer (CRC) accounts for over 25% of all cancer-related deaths with global incidence rates steadily rising [1–3]. Since less than 15% of CRC risk has been attributed to heritable genetics [4, 5], non-shared exposures and their contributions to gut inflammation are believed to be important etiologic factors [6]. Increased CRC risks have been associated with cigarette smoking, alcohol use, lack of physical activity, obesity, abnormal glucose metabolism, and consumption of red meat and n-6 polyunsaturated fatty acids (PUFAs) [7–10]. Conversely, consumption of n-3 PUFAs, fruits, fish, vitamins D and E, and regular use of aspirin appear to reduce CRC risks [7, 11, 12]. There are also persistent suggestions that the interplay between dietary factors - particularly red meat, lipids, and fiber - and the gut microbiota are effect modifiers for CRC [6, 13–16].

Many of the associations between exposures and CRC have been gleaned from epidemiological studies that employed self-reported dietary and lifestyle factors [8, 9, 16, 17]. Given the inherent limitations of such data for discovering causal exposures, investigators have recently employed metabolomics to compare small-molecule features between CRC cases and controls. This strategy is based on the idea that small molecules in human blood reflect chemical exposures from both internal and external sources, including the diet, microbiota, psychosocial stress, and pollutants [18]. However, since molecules that discriminate cases from controls in cross-sectional studies can reflect both potential causes of CRC and dysregulation of metabolic processes that result from progression of the disease (reverse causality) [5, 19], it is important that biospecimens be collected before diagnosis to gain insights into causes and effects. Indeed, a class of ultra-long-chain fatty acids (ULCFAs) that discriminated for CRC in several cross-sectional studies [20, 21] was essentially ruled out as a causal factor in a prospective cohort [22].

Metabolomic analyses of blood from prospective cohorts have found some associations between CRC incidence and small molecules, as summarized in Table 1, with periods of follow-up ranging from 3.7 to 14.7 years [19, 22–27]. Interestingly, all of these nested case-control studies followed targeted or semi-targeted designs where relatively few molecular features were tested between cases and controls. Two of the studies focused on metabolism of dietary choline and found that the mammalian metabolite, betaine, was moderately protective against CRC whereas trimethylamine-*N*-oxide (TMAO), a metabolite mediated via intestinal microbiota, was associated with increased risk [26, 27]. A genetic link between TMAO and CRC risk has also been reported [28]. Intriguingly, red meat and other phosphatidylcholine-rich foods appear to contribute to dysbiotic microbiota that generate trimethylamine (the precursor of TMAO) [15, 29], whereas fiber-rich foods appear to encourage symbiotic bacteria that are associated with decreased CRC risk [15, 30].

**Table 1:** Studies that investigated associations of colorectal cancer with small molecules in plasma or serum from prospective cohorts

Cohort	Cases/Controls	Follow-up^a^ (y)	Analytical method	Design	Exposure variable	Likely associations	Ref.
WHI-OS	835/835	5.2	LC-MS	Targeted	Choline and its metabolites	TMAO (+); betaine/choline ratio (−)	[26]
EPIC [1]	1367/2323	3.7	LC-MS	Targeted	Methionine and choline metabolites	Methionine, choline, and betaine (−)	[27]
EPIC [2]	95/95	14.7	LC-MS	Targeted	8 Ultra-long-chain hydroxylated fatty acids	All associations (−) diminished with time to diagnosis (reverse causality)	[22]
EPIC [3]	1238/1238	3.8	Colorimetry and turbidimetry	Targeted	Triglycerides, cholesterol, and lipoproteins	HDL (−)	[19]
PLCO	254/254	7.8	LC-MS and GC-MS	Semi-targeted	278 Annotated metabolites detected in > 80% of specimens	Glycochenodeoxycholate (+) in women but not men	[25]

*WHI-OS* Women’s Health Initiative Observational Study, *EPIC* European Prospective Investigation into Cancer, *GC-MS* gas chromatography-mass spectrometry, *HDL* high-density lipoprotein cholesterol, *LC-MS* liquid chromatography-mass spectrometry, *PLCO* Prostate, Lung, Colorectal, and Ovarian Cancer Screening Trial, *TMAO* trimethylamine-*N*-oxide, *WHI-OS* Women’s Health Initiative-Observational Study, (+) positively associated with CRC, (−) negatively associated with CRC

^a^Mean period of follow-up

Since untargeted metabolomics via liquid chromatography-mass spectrometry (LC-MS) can detect thousands of small-molecule features, traditional hypothesis-testing approaches that adjust for multiple comparisons by controlling false positive error rates, such as the false discovery rate (FDR) [31], can make it difficult to find features whose levels differ significantly between cases and controls. This may have motivated the semi-targeting strategy of Cross et al. (Table 1) [25], who limited hypothesis tests of the thousands of detected features to only 278 molecules that had been fully annotated. Such a strategy is likely to be biased towards well curated metabolites that participate in recognized human pathways [18], and thus can miss novel exposures of potential importance to initiation of cancer, including those experienced predominately by either cases or controls. Indeed, of the 278 small-molecules tested by Cross et al. [25], only glycochenodeoxycholate (a secondary bile salt) was associated with increased CRC risk in women (but not men) after using the conservative Bonferonni correction of the *p*-value.

Here, we report results of an untargeted metabolomics analysis of serum from 66 incident CRC cases and matched controls from the European Prospective Investigation of Cancer and Nutrition (EPIC). Given the involvement of lipids in inflammatory processes and CRC [32–34], the serum-extraction procedure favored lipophilic molecules. As an alternative to the traditional multiple-hypothesis-testing paradigm for selecting features of potential importance to CRC, we developed a variable-selection strategy that employs an ensemble of diverse prediction methods, including regularized linear regression and regression trees [35–37]. Such methods have recently been applied independently for analyzing metabolomic and other -omic data [35, 36, 38]. Our analyses point to a small set of features that were predictive of CRC-case status. However, as with all discovery studies, these potentially important features and the molecules they represent must be further validated with independent data sets.

## Methods

### Study population

EPIC is a prospective cohort study with approximately 520,000 adult participants from across Europe that were enrolled from 1992 through 2000 [39]. We had received EPIC serum to test the hypothesis that ULCFAs were protective of colorectal cancer [22], and simultaneously performed untargeted metabolomics to discover other potentially causal features. For the current study, data were analyzed from serum collected between 1993 and 1997 from 66 case-control pairs in Turin, Italy. Controls were matched to incident cases by age, year and season of enrollment, and gender. Dietary data were collected with food frequency questionnaires [40, 41]. Summary statistics for these subjects are listed in Table 2, including time to diagnosis (*ttd*), gender, body mass index (*bmi*), waist circumference, smoking status, diabetes status, physical activity, and alcohol and meat consumption. These variables were selected based on previous evidence of associations with CRC risk [7, 42, 43]. Across our subjects, the only significant differences between CRC cases and controls were observed for *bmi* and waist circumference, both of which were higher in cases (Table 2).

**Table 2:** Descriptive statistics of human subjects matched by age, study enrollment, gender, and selected covariates

	Total *n*=132	CRC cases *n*=66	Controls *n*=66	*p*-value^*^
Gender	Male	51	51	
	Female	15	15	
Age at enrollment (y)	median	56	56	
	min	35	35	
	max	65	65	
Years to diagnosis	median	7.52	–	
	min	0.10	–	
	max	14.40	–	
BMI	median	26.9	25.3	0.0246
	min	19.7	18.7	
	max	36.7	33.6	
Waist circumference (cm)	median	97	90	0.0016
	min	68	66	
	max	115	119	
Diabetes	yes	2	2	
	no	64	64	
Smoking Status	current	15	16	0.8423
	former	27	23	
	never	21	22	
	NA	3	5	
Alcohol consumption (ml/day)	median	23.0	22.6	0.4038
	min	0.0	0.1	
	max	79.8	113	
Physical Activity (min/day)	high	13	13	0.6102
	medium	15	20	
	low	25	18	
	none	10	10	
	NA	3	5	
Total meat consumption (g/day)	median	75.6	67.6	0.4640
	min	5.9	8.8	
	max	248.3	201.3	

*NA* not available

^*^Nominal *p-*values calculated from two-sided Wilcoxon rank sum tests (dichotomous variables) or chi-square tests (categorical or continuous variables)

### Chemicals

Isopropanol (LC-MS grade, Fluka), methanol, water and ^13^*C-* cholic acid (internal standard) were from Sigma-Aldrich (Milwaukee, WI, USA). Acetic acid (LC-MS grade, Optima) and chloroform were from Fisher Scientific (Santa Clara, CA, USA). All chemicals were of analytical grade and were used without purification.

### Sample processing

Serum was stored after collection in 0.5-ml aliquots that were placed in cryostraws, sealed, and stored in liquid nitrogen (-196 °C) at the International Agency for Research on Cancer in Lyon, France. Approximately 1 year prior to analysis, cryostraws were transported (with dry ice) to our laboratory in Berkeley, CA (USA), where they were maintained at -80 °C. As previously reported [22], 20 μl of serum was mixed with 100 μl of a solvent mixture (isopropanol/methanol/water = 60:35:5) containing ^13^*C*-cholic acid as an internal standard (final concentration of 3.0 μg/ml). After mixing samples for 1 minute with a vortex mixer, samples were left at room temperature for 10 min. to precipitate proteins, and were then centrifuged for 10 min at 10,000 g. The supernatant was retained and stored at 4 °C prior to LC-MS. Case-control pairs were analyzed sequentially but in random order. A local quality-control sample, prepared by pooling aliquots from all serum specimens of each batch, was analyzed after every ten samples to monitor system stability and estimate the precision of the analyses.

### Mass spectrometry

Analysis was performed with an Agilent LC (1100 series) coupled to an Agilent high resolution MS (Model 6550 QTOF, Santa Clara, CA, USA) as previously reported [22]. Briefly, 10 μl of extracts were slowly loaded on to a Luna C5 column (Phenomenex, Los Angeles, CA) with a 22-min gradient elution of mobile phase A (methanol/0.5% acetic acid = 5:95) and mobile phase B (isopropanol/methanol/0.5% acetic acid = 60:35:5). The electrospray was operated in negative electrospray-ionization (ESI) mode. Tandem MS/MS spectra were obtained on the same platform in data-dependent mode (immediately after data collection) or targeted mode (analysis of the selected features). Full LC-MS acquisition parameters were previously published [22].

Approximately one third of the serum samples had a gelled consistency that resulted from an additive to the cryostraws [22, 44, 45]. Pairs with at least one gelled sample were analyzed in one batch (batch 1, *n* = 96), and the remaining (non-gelled) pairs were analyzed in a second batch (batch 2, *n* = 36).

### Data processing

Raw data were converted to mzXML format for peak picking using ProteoWizard software (Spielberg Family Center for Applied Proteomics, Los Angeles, CA). Peak detection and retention-time alignment were performed as described previously [22], using the XCMS package within the R statistical programming environment [46–48]. The CAMERA package was used to identify isotopes, ESI adducts, and in-source fragments with the custom rule set used from Stanstrup et al. [49, 50]. Annotation of features was conducted using the compMS2Miner package [51], by comparing accurate masses and MS2 fragmentation patterns with the Human Metabolome Database (HMDB) and Metlin [52, 53].

Over 24,300 features were initially detected in negative ESI mode. Features were filtered by removing those with a mean fold-change in abundance less than 1.5 compared to the same peaks in reagent blanks (background noise) and those with coefficients of variation (CV) from QC samples greater than 30% [54, 55]. This resulted in a final dataset of 8690 features for statistical analysis. Feature intensities were (natural) log-transformed and adjusted for batch and gel-status effects using the following linear regression model, previously described in [22]:

1 $$ \mathit{\log}{Y}_i={\beta}_0+{\beta}_1{X}_{i, gel}+{\beta}_2{X}_{i, batch}+{\epsilon}_i, $$

where *Y*_*i*_ denotes the intensity of a given feature for the *i*^th^ subject and *X*_*i*, *gel*_ and *X*_*i*, *batch*_ are the corresponding categorical covariates for gel-status and batch. After fitting the linear model, normalized (logged) intensities were obtained by subtracting the estimated batch and gel effects from the original (logged) intensities.

Upper-quantile scaling was used to render the distributions of feature abundances more comparable across all subjects [56, 57]. A correlation-network program (Cytoscape, [58]) and an R package clustering algorithm (RAMclust, [59]) were used to identify clustered ions and assist with annotations.

### Statistical methods: Variable selection

In order to identify discriminating features between CRC cases and control, we shifted the paradigm from multiple hypothesis testing to variable selection based on a combination of three regression methods. First, we considered the following standard linear regression model for the raw intensity of a given feature *Y* in the *i*^th^ subject:

2 $$ \mathit{\log}{Y}_i={\beta}_0+{\beta}_1{X}_{i, caco}+{\beta}_2{X}_{i, gel}+{\beta}_3{X}_{i, batch}+{\beta}_4{X}_{i, age}+{\beta}_5{X}_{i, gender}+{\epsilon}_i, $$

where *caco, gel,* and *gender* denote binary variables for case-control status, presence or absence of gelled serum, and the matched variables of *gender* and *age* (in years). Features were then ranked based on the nominal unadjusted *p*-value for the case-control coefficient (*β*_1_). Second, a regularized logistic regression (LASSO: least absolute shrinkage and selection operator) was performed [35, 60] with case-control status as the binary outcome variable regressed on the following covariates: normalized log intensities from Eq. (1) for all 8690 metabolites, *age* and *gender*. Although *age* and *gender* were in the LASSO model, neither variable was selected by the regularized regression.

In order to stabilize feature selection with LASSO, 500 bootstrap samples were taken for each of a variety of penalty parameters [61]. Features chosen by LASSO in at least 10% of the bootstrap samples, across a wide range of penalty parameters, were retained. A data-driven cutoff of 10% was chosen based on plots of the percentage of time that each metabolite was selected during the bootstrap iterations across various penalty parameters, sorted in decreasing order. There was an obvious gap between metabolites selected more than and less than 10% of the time, which lead to choosing this as a natural cutoff. Third, the random forest algorithm [36, 62] was used to build a predictor of case-control status, using the same covariates as for the LASSO regression. No obvious jump in variable importance could be seen for the sorted random forest variable importance or the sorted linear regression *p*-values. Therefore, a cutoff of 1% was selected for both of these criteria because this cutoff is relatively stringent, yet still included a reasonable number of variables for consideration. In summary, to select a final set of variables, we included only features that were selected by the bootstrap LASSO and were also among the top 1% of features ranked by linear regression *p*-values and random forest variable importance.

When a set of features was selected that satisfied all criteria, the extracted ion chromatographs were visually inspected and those with poor peak morphology (ill-defined Gaussian shape) or integration were removed. Then, the three variable selection methods were repeated as needed to arrive at a final set of selected features with good peak morphology and integration.

Initially, only covariates on which the samples were matched (i.e. *age* and *gender*) were included in the models used for variable selection. We did not include other dietary or health related covariates because we did not want to obscure possible associations between the metabolites and case-control status. However, we did subsequently test for associations between the nine selected metabolites and the following covariates *weight, bmi, smoking status, and consumption of beef, pork, and alcohol*. As shown in Additional file 1, only one feature (ID 839) was marginally associated with any of the covariates (i.e. *bmi* and *consumption of beef*). Also, when each of these covariates was added to the LASSO model (Eq. (2)) with case-control status as the binary outcome variable none of them was selected by the regularized regression.

## Results

### Features that discriminate for CRC

**Fig. 1:**
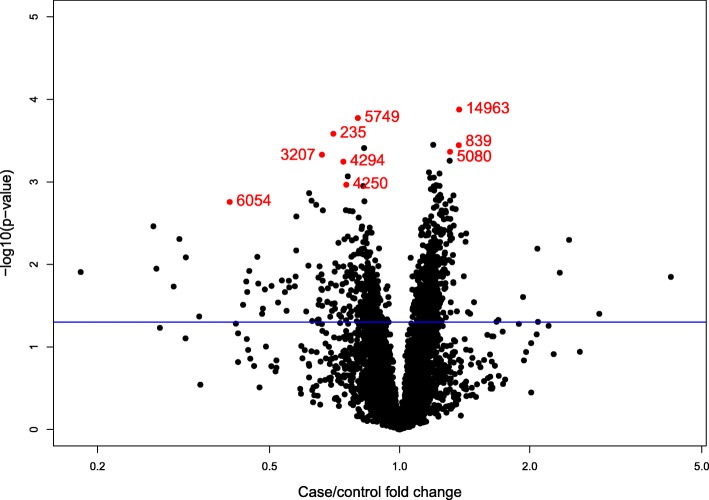
Volcano plot of analyzed features; the nine selected features are highlighted in red with the ID labels from Table 3. An arbitrary *p*-value = 0.05 threshold line is present for reference. *p*-values and fold-changes are calculated based on the regression model in Eq. (2)

After applying the three variable-selection methods described above, two of which prioritize predictive ability (LASSO and random forest), nine features were selected. The volcano plot in Fig. 1 relates case-control fold changes and –log_10_ *p*-values (for the model in Eq. 2) for all features and highlights the nine selected metabolites (shown in Table 3). Case-control fold-changes ranged from approximately 0.2 to 3.0 overall and between 0.40 and 1.40 for the nine selected features. Due to the nature of our variable selection method, the *p*-values of the selected features were not necessarily the smallest, nor were their fold-changes necessarily the largest. Nevertheless, the nine selected metabolites resulted in a 79% correct classification rate when they were used to fit a logistic regression model on the learning set to predict case-control status. Although this correct classification is likely optimistic because the same data were used to perform the variable selection and to build and test the predictor, the selected features are worthy of validation in independent samples of CRC cases and controls from prospective cohorts.

**Table 3:** Untargeted features selected as predictors of case-control status

Feature ID	Observed *m/z* ^a, b^	Ret. time (sec)	Fold change	*p*-value	Feature type ^c^
235	391.2832	596.4	0.702	0.000261	R
4250	453.3592	605.5	0.753	0.001082	R
4294	467.3744	605.6	0.741	0.000569	R
5080	519.1965	595.9	1.308	0.000432	C
3207	531.1558	563.9	0.661	0.000468	C
6054	551.1781	563.9	0.404	0.001750	C
839	577.2698	620.6	1.370	0.000359	C
5749	882.6393	718.2	0.880	0.000168	I
14,963	907.4806	617.6	1.373	0.000133	R

*m/z* mass-to-charge ratio, *p*-value from the regression model (Eq. 2), *C* potentially causal feature, *R* potentially reactive feature, *I* indeterminate

^a^Observed *m/z* values correspond to singly-charged negative ions

^b^Feature selected by bootstrap LASSO and by being in the top 1% of features ranked by both the *p*-values from the case-control regression (Eq. 2) and the random forest variable importance measure

**Fig. 2:**
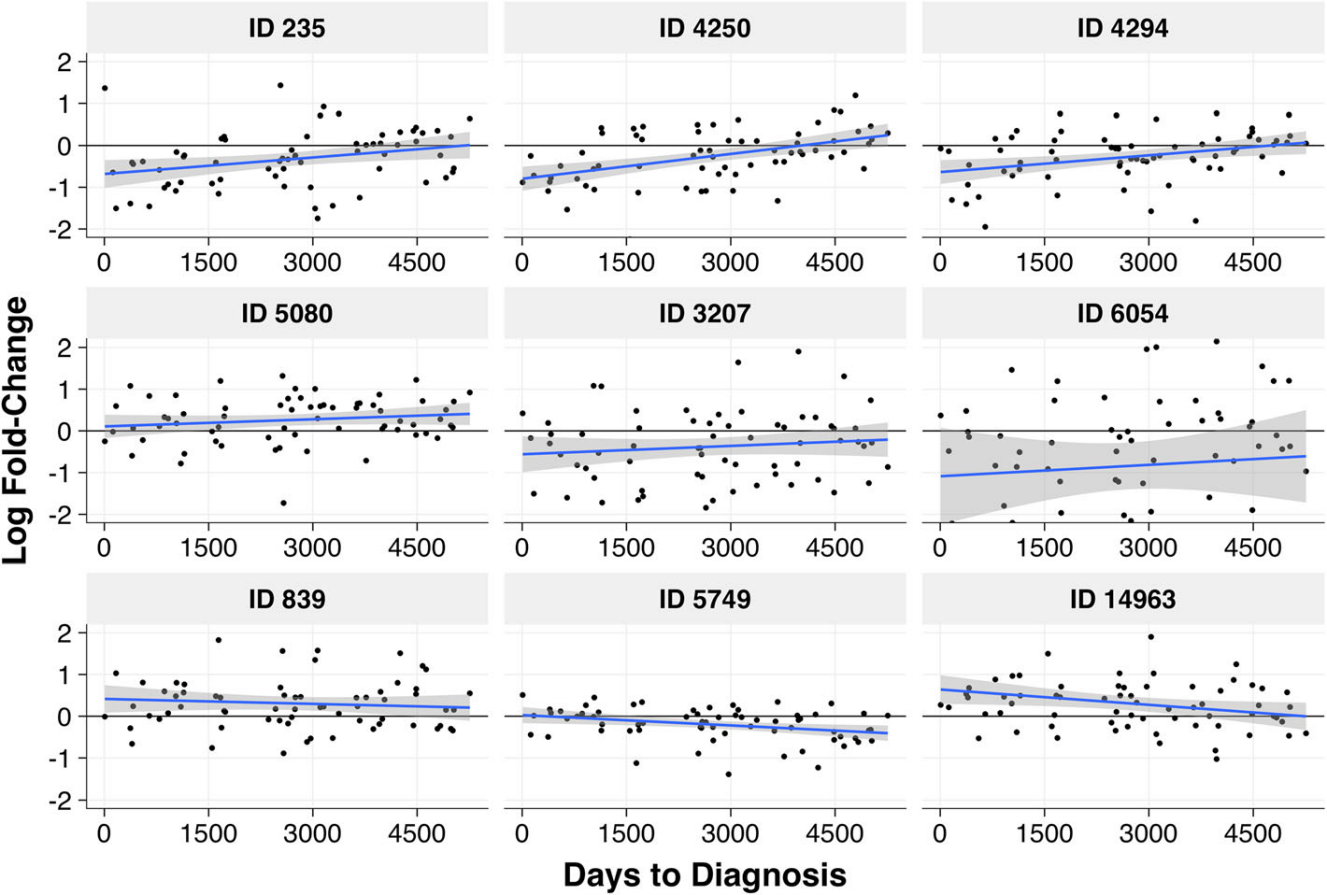
Scatterplots of case-control log fold-change vs. time to diagnosis (*ttd*) for the 9 selected features. The blue line is the linear regression fit and the gray band represents a 95% confidence intervals, calculated with the ‘lm’ method of the R function ‘geom_smooth’ in the package ‘ggplot2’

^c^Based on regression of case-control difference on time to diagnosis (*ttd*, Fig. 2)

### Potentially causal and reactive biomarkers

The nine selected features were evaluated to determine their associations with time to diagnosis (*ttd)* as a means of discerning whether they represent potentially causal exposures or reactive effects of disease progression [22]. If the log fold-change for a given feature was constant across the whole range of *ttd* in a linear model (*p*-value > 0.05), the feature was classified as potentially causal (C) and if the case-control difference decreased with increasing *ttd*, the feature was classified as potentially reactive (R). These (C) and (R) classifications are listed in Table 3 for the nine selected features and the plots for the *ttd* linear models are shown in Fig. 2. (See Additional file 1 for regression coefficients and *p*-values). This process resulted in four potentially causal features (no apparent effect of *ttd* for IDs: 5080, 3207, 6054 and 839), four potentially reactive features (case-control differences diminish with *ttd* for IDs: 235, 4250, 4294 and 14,963), and one feature that could not be classified as either (C) or (R) (case control differences increased with *ttd*, ID 5749). The four potentially causal metabolites resulted in a 72% correct classification rate to predict case-control status (with the same caveats mentioned above). While the four potentially causal features may be linked to exposures that contribute to CRC, the four reactive features may be useful pre-diagnostic biomarkers.

## Discussion

Using untargeted metabolomics in serum samples from 66 pairs of CRC cases and controls from the EPIC cohort, we sought evidence linking lipophilic molecules with the etiology of CRC. The LC-MS data collected from these samples included over 24,000 features. After filtering for noise (mean fold-change above blank samples), reproducibility (CVs), and likely artifacts (CAMERA), 8690 features were available for evaluating potential associations with CRC case status.

It has been standard practice in metabolomics to identify features that discriminate for case-control status using a multiple-testing approach, e.g., based on a cutoff for *p*-values that have been adjusted to control for a false positive error rate such as the FDR [63]. Since untargeted metabolomics can detect thousands of features, FDR correction is severe [63] and can drastically reduce the number of selected metabolites, thereby resulting in false negatives. Thus, we shifted our paradigm to a variable-selection approach, based on an ensemble of diverse regression methods, in order to uncover a reliable set of features for further investigation. This led to selection of 9 features that discriminated for CRC (Table 3) with a correct classification of 79%. Based on regressions of case/control fold changes on *ttd* (Fig. 2), four of these features appear to be related to causal factors for CRC, four appear to be related to cancer progression, and one is indeterminate.

As mentioned earlier, the top 1 % of features in terms of smallest *p*-values were considered for variable selection. However, many of these roughly 90 features were not also high ranking in terms of predictive importance, as assessed by random forest and LASSO. Indeed, inspection of data from the features with very small *p*-values found some to have small standard errors rather than meaningful case-control differences, while other features with large fold changes had great uncertainties in fold-change estimates. This reinforces the value of using an ensemble of regression methods to evaluate biological variability rather than a single measure such as a small *p*-value or a large case-control fold change.

### Possible annotations

Potential annotations of the nine selected features were based on comparisons of MS2 spectra with human metabolome database (HMDB) entries as summarized in Table 4. Focusing first on the four potentially causal features shown in Table 3, MS2 were only obtained for IDs 3207 and 6054, which were positively correlated (Pearson correlation coefficient of 0.64) and were both present at lower levels in cases than in controls, indicating possibly protective effects. The two MS2 fragment ions detected for ID 3207 could not be identified. However, ID 6054 had fragments characteristic of [C_16_H_29_O_2_]^−^ and losses of two H_2_O molecules, consistent with the loss of two hydroxyl groups and a hexadecenoic acetate fragment. These fragments are suggestive of ceramide lipids [64], a class of molecules that has been implicated in the regulation of cancer cells [65]. Although IDs 3207 and 6054 and the two other potentially causal features (IDs 5080 and 839) could not be fully annotated, the identified characteristics of accurate mass, retention time, and MS2 fragments can be used for validation in future studies.

**Table 4:** Results of tandem MS/MS analyses of features associated with case-control status

Feature ID	Observed *m/z*	Ret. time (s)	Prominent MS2 fragments with possible fragment IDs	Putative ID	Species	Molecular formula	Δ ppm
235	391.2832	596.4	347.2961(loss of CO_2_), 197.0725 ([C_10_H_13_O_4_]-)	possible fatty acid	[M-H]-	C_24_H_40_O_4_	4.22
4250	453.3592	605.5	59.0131, 409.3687 (loss of CO_2_), 391.3568 (loss of CO_2_ and H_2_O), 279.2336 ([C_18_H_31_O_2_]-), 435.3462 (loss of H_2_O)	possible ULCFA	[M-H]-	C_27_H_50_O_5_	−2.76
4294	467.3744	605.6	449.3639 (loss of H_2_O), 263.2368 ([C_18_H_31_O]-), 423.3842 (loss of CO_2_), 162.8392, 405.3724 (loss of CO_2_ and H_2_O)	ULCFA 468	[M-H]-	C_28_H_52_O_5_	−1.56
5080	519.1965	595.9	No MS2 spectra	unknown			
3207	531.1558	563.9	481.3110, 256.2357	unknown			
6054	551.1781	612.3	478.2903, 515.1326 (loss of 2 H_2_O;), 253.2165 ([C_16_H_29_O_2_]-)	possible ceramide			
839	577.2698	596.8	No MS2 spectra	unknown			
5749	882.6393	718.2	124.0075, 822.6453 (loss of acetate)	unknown	[M + HAc-H]-		
14,963	907.4806	617.6	No MS2 spectra	unknown			

Turning now to the likely reactive features, ID 4294 was putatively identified as ULCFA 468, which had been evaluated separately in our targeted study of 8 ULFCAs [22] and had been first reported by Ritchie et al. [24]. This feature had neutral losses of H_2_O and CO_2_, characteristic of a hydroxylated fatty acid and a likely molecular formula for [M-H]^−^ of C_28_H_52_O_5_, within 1.56 ppm of the exact mass. Another reactive feature (ID 4250) also displayed these characteristic neutral losses of H_2_O and CO_2_ and was highly correlated with ID 4294 with a correlation coefficient of 0.85. This suggests that ID 4250 is a previously uncharacterized ULCFA with molecular formula for [M-H]^−^ of C_27_H_50_O_5_, within − 2.76 ppm. While odd-numbered fatty acids are less common in humans, microbial single carbon metabolism in very long chain fatty acids has been reported [66]. As a class, ULCFAs tend to be present at higher levels among controls compared to paired cases, but this difference diminishes with *ttd*, suggesting that they result from disease progression [22]. Nonetheless, the fact that these two ULCFAs were selected from approximately 9000 features that survived filtering of the untargeted metabolomics data offers partial validation to our variable-selection strategy. Based on correlation maps (data not shown) both of these features clustered with five other ULCFAs that have been described by Ritchie, et al. [24] (ULCFAs 465, 466, 492, 518, and 538; exact masses within 10 ppm of calculated *m/z*), and were also analyzed in our targeted study [22]. Another selected feature (ID 235) exhibited similar reactive (R) behavior to the ULCFAs (Fig. 2), and the presence of a neutral loss of CO_2_, indicating that ID 235 may be a fatty or bile acid. Deoxycholic acid (3α, 12α-dihydroxy-5β-cholanic acid) [M-H]^−^, chenodeoxycholic acid (3α, 7α-dihydroxy-5β-cholanic acid) [M-H]^−^, and adrenic acid [M + HAc-H]^−^ were eliminated as possible annotations of feature 235 by comparison of retention times between the experimental data and analytical standards. However, these two tested molecules are just two isomers of a large class of bile acids, some of which are positively correlated with CRC [25, 67]; in our study, feature 235 was negatively correlated with CRC.

### Limitations

Limitations of this study include the small sample size, which reduced the power to detect differences between case-control pairs, and lack of information regarding aspirin consumption and a family history of CRC, two covariates that have been associated with CRC incidence [1, 68]. Any bias (and potential confounding) introduced by the gelling of some samples from the cryostraws should have been removed by adjustment via Eq. (1). Nonetheless, gelling complicated sample processing and was, therefore, a source of random variation that probably reduced our ability to detect differences between cases and controls. Gelling of EPIC serum resulted from a ‘gel powder’ that had been added to seal one end of the cryostraw (https://patents.google.com/patent/US7056727B2/en). This illustrates how proper storage of biological specimens for decades is challenging because preservation of cells, DNA, RNA, proteins and small molecules must be considered. However, decades later, shortcomings of then-contemporary technology (such as gelling of serum) can be revealed and their reporting can improve the design of future investigations.

## Conclusions

In summary, of the nearly 9000 filtered features subjected to statistical analysis, four appear to be potentially causal features that are worthy of following up in an independent set of prospective CRC cases and controls. When these four features alone were used to build a logistic regression predictor of case/control status on the learning set, they resulted in a correct classification rate of 72%. Again, this is likely an optimistic correct classification rate, but given that only four features were used for prediction, it is quite promising. Four other selected features, notably some ULCFAs and related fatty acids, appear to be products of disease progression and, therefore, could be useful diagnostic biomarkers for early detection of CRC. Since ULCFAs had previously been shown to discriminate CRC cases from controls in several cross-sectional investigations, it is reassuring that two putative ULCFAs (IDs 4294 and 4250) were selected as predictive features in this untargeted analysis. While the relatively modest number of samples limited the power to detect other associations, the nine features selected in our study correctly predicted case-control status in 79% of the samples. The stability of these features across three disparate feature-selection methods is promising. Furthermore, based on *m/z* and annotation information, these nine features appear to be different than those reported in the prospective CRC study by Cross et al. [25], warranting further identification and validation.

## Additional file

Additional file 1: Additional statistical analyses of the nine selected metabolomic features. **Table S1.** Covariate associations with the nine selected features; **Table S2.** Time-to-diagnosis model coefficients and *p*-values for the nine selected features. (PDF 60 kb)

## Abbreviations

bmi: Body mass index
CRC: Colorectal cancer
CV: Coefficient of variation
EPIC: European prospective investigation into cancer and nutrition
FDR: False discovery rate
HMDB: Human Metabolome Database
LASSO: Least absolute shrinkage and selection operator
LCMS: Liquid chromatography mass spectrometry
PUFA: Poly-unsaturated fatty acid
TMAO: Trimethylamine-*N*-oxide
*ttd*: Time to (case) diagnosis
ULCFA: Ultra-long chain fatty acid
