# Supplementary material for: Untargeted lipidomic features associated with colorectal cancer in a prospective cohort
Source: BMC Cancer. 2018 Oct 19;18:996. doi: 10.1186/s12885-018-4894-4 (PMC6194742; doi:10.1186/s12885-018-4894-4)
Supplement: Supplementary file 1 — Additional statistical analyses of the nine selected metabolomic features. Table S1. Covariate associations with the nine selected features; Table S2. Time-to-diagnosis model coefficients and p-values for the nine selected features. (PDF 60 kb) [file 12885_2018_4894_MOESM1_ESM.pdf]

Additional File 1

Lipidomic features associated with colorectal cancer in a prospective cohort

Perttula, Kelsi[a]; Schiffman, Courtney[a]; Edmands, William MB[a]; Petrick, Lauren[a,g]; Grigoryan, Hasmik[a]; Cai, Xiaoming[a]; Gunter, Marc J[c]; Naccarati, Alessio[d], Polidoro, Silvia[d], Dudoit, Sandrine[a,b,f] ; Vineis, Paolo[d,e]; Rappaport, Stephen M[a]*

BMC Cancer 2017

[a] School of Public Health, University of California, Berkeley, California, 94720, United States
[b] California Institute for Quantitative Biosciences, University of California, Berkeley, California 94720, United States
[c] International Agency for Research on Cancer, Lyon, France
[d] Italian Institute for Genomic Medicine (IIGM), Torino, Italy
[e] MRC-PHE Centre for Environment and Health, Imperial College, Norfolk Place London W2 1PG, UK
[f] Department of Statistics, University of California, Berkeley, CA, United States
[g] Department of Environmental Medicine and Public Health, Icahn School of Medicine at Mount Sinai, New York, NY
* Corresponding author: srappaport@berkeley.edu

Additional Table 1: Covariate associations with the nine selected features.

Feature ID	Coef-weight	*p*-value-weight	Coef-former smoker	*p*-value-former smoker	Coef-never smoker	*p*-value-never smoker	Coef-dietary beef	*p*-value-dietary beef	Coef-dietary pork	*p*-value-dietary pork	Coef-alcohol	*p*-value-alcohol	Coef bmi	*p*-value-bmi
235	-0.0071	0.1213	-0.0101	0.9340	-0.0827	0.5079	-0.0001	0.9229	0.0002	0.9655	-0.0015	0.5219	-0.0248	0.1112
4250	-0.0078	0.0601	-0.0465	0.6722	0.0262	0.8165	-0.0002	0.8705	-0.0006	0.8929	-0.0013	0.5335	-0.0206	0.1374
4294	-0.0043	0.2984	-0.1261	0.2487	-0.0136	0.9031	-0.0003	0.8180	0.0021	0.6509	-0.0009	0.6599	-0.0105	0.4553
5080	-0.0022	0.5452	0.0651	0.4973	-0.0002	0.9981	-0.0004	0.7031	0.0068	0.0929	0.0016	0.3770	-0.0081	0.5092
3207	-0.0041	0.4651	-0.0045	0.9759	0.0505	0.7412	0.0009	0.5996	-0.0006	0.9255	0.0001	0.9800	-0.0213	0.2582
6054	0.0009	0.9485	0.2245	0.5394	0.3225	0.3907	-0.0013	0.7523	0.0065	0.6759	-0.0034	0.6284	-0.0380	0.4185
839	0.0052	0.2182	0.0926	0.4019	-0.0537	0.6354	0.0024	0.0514	0.0025	0.6037	0.0009	0.6842	0.0315	0.0258
5749	-0.0016	0.5706	0.0211	0.7730	0.1283	0.0899	0.0001	0.9387	-0.0042	0.1860	-0.0008	0.5798	-0.0145	0.1267
14963	-0.0009	0.8265	0.0334	0.7481	-0.0352	0.7413	0.0000	0.9714	-0.0065	0.1394	-0.0007	0.7207	0.0034	0.8024

Additional Table 2: Time-to-diagnosis model coefficients and *p*-values for the nine selected features.

Feature ID	Coefficient- intercept	*p*-value- intercept	Coefficient- *ttd*	*p*-value- *ttd*
235	-0.683279638	0.000114227	0.000131033	0.017420508
4250	-0.797035644	6.42E-07	0.000197727	7.21E-05
4294	-0.638321519	3.04E-05	0.000133492	0.005003649
5080	0.105665952	0.457263603	5.65E-05	0.220430648
3207	-0.561619075	0.011411263	6.62E-05	0.345379446
6054	-1.088369097	0.065831603	9.06E-05	0.631118813
839	0.412131079	0.015836933	-3.97E-05	0.462480298
5749	0.024339434	0.801178971	-8.29E-05	0.009654109
14963	0.638445593	0.000486145	-0.000122715	0.032348755

ttd- time to diagnosis
